# Mesogen-Containing Reactive Epoxy Monomer for Tuning the Thermal, Rheological, and Mechanical Properties and Fracture-Surface Morphology of Thermally Conductive Epoxy Potting Compounds

**DOI:** 10.3390/polym18121503

**Published:** 2026-06-16

**Authors:** Huize Cui, Ruilu Guo, Chong Zhang, Hui Liu, Xiaoxuan Liu, Jinyan Wang, Xigao Jian

**Affiliations:** 1 State Key Laboratory of Advanced Power Transmission Technology (China Electric Power Research Institute Co., Ltd.), Beijing 102209, China; jameschz@126.com (H.C.); grl18810663757@163.com (R.G.); 18611602136@163.com (C.Z.); 18618129466@163.com (H.L.); 2 State Key Laboratory of Fine Chemicals and Department of Polymer Science and Engineering, Dalian University of Technology, Dalian 116024, China; liuxiaoxuan6021@163.com (X.L.); jian4616@dlut.edu.cn (X.J.); 3 Liaoning Province Engineering Research Centre of High-Performance Resins, Dalian 116024, China

**Keywords:** mesogenic unit, reactive epoxy monomer, thermally conductive potting compound, rheological behavior, glass transition temperature, fracture morphology

## Abstract

Thermally conductive epoxy potting compounds require high filler loadings for effective heat dissipation. However, high filler loadings can increase viscosity and brittleness, thereby impairing processability and service reliability. In this study, a mesogen-containing reactive liquid–crystalline epoxy monomer (LCE) was designed, synthesized, and incorporated into a commercial thermally conductive epoxy potting compound to investigate its effects on thermal behavior, rheological and mechanical properties, thermal conductivity, and fracture-surface morphology. The chemical structure and thermotropic liquid–crystalline behavior of LCE were characterized via Fourier-transform infrared spectroscopy, proton nuclear magnetic resonance spectroscopy, differential scanning calorimetry, and polarized optical microscopy. Increasing LCE loading elevated the DSC-derived glass transition temperature (T_g_) from 59 °C to 96 °C and markedly increased the room-temperature complex viscosity. Single-point measurements at 25 °C showed a monotonic decrease in thermal conductivity from 0.95 to 0.52 W/(m·K) with increasing LCE content. Mechanical testing revealed that the nominal 10% LCE formulation provided the best balance between load-bearing capacity and ductility among the tested formulations, whereas higher LCE loadings were associated with greater local microstructural variation and reduced mechanical properties. This study clarifies the modulation effect of LCE on the performance balance of highly filled epoxy potting compounds, providing valuable insights for future formulation optimization.

## 1. Introduction

The rapid development of 5G communication systems, new energy vehicles, and high-power electronics has driven a continuous increase in the integration and power density of related electronic devices [1,2,3]. In this context, heat accumulation has become a critical bottleneck restricting the operational stability and long-term reliability of these devices: prolonged thermal buildup accelerates material degradation, induces thermal stress, and ultimately leads to performance deterioration or even catastrophic device failure [4,5,6]. Accordingly, efficient thermal management has become an indispensable requirement for state-of-the-art electronic packaging technologies [7,8,9]. Among various thermal management solutions, thermally conductive packaging and potting materials play a pivotal role. These materials not only enable efficient heat dissipation from localized heat sources but also provide robust electrical insulation and mechanical protection for core devices [10,11]. For practical deployment, such materials must simultaneously possess high thermal conductivity, excellent thermal and dimensional stability, and good processability. However, balancing these comprehensive properties remains a long-standing challenge, especially for resin-based potting systems used in complex encapsulation scenarios [12,13].

Epoxy resins are one of the most widely used polymer matrices for electronic packaging and potting applications, owing to their outstanding adhesion, excellent electrical insulation, high dimensional stability, and tunable curing processability [14,15,16]. Commercial thermally conductive epoxy potting compounds are particularly popular in industrial scenarios, as they combine scalable manufacturing processability with reliable electrical insulation and dimensional stability [17,18]. Nevertheless, achieving a well-balanced performance among thermal dissipation capabilities [19], rheological behavior [20], thermomechanical stability, and long-term mechanical reliability [21] remains a key bottleneck for highly filled commercial epoxy potting systems, severely limiting their application in demanding high-power electronic fields.

Current strategies to address this challenge mainly fall into two well-established categories. The first is filler engineering, including the rational design of hybrid filler systems and the construction of segregated or interconnected thermal conduction networks. For instance, this approach has been implemented via the fabrication of boron nitride/alumina hybrid filler systems or three-dimensional (3D) continuous thermal transfer pathways [22,23,24]. While this strategy effectively improves thermal transfer efficiency, it cannot fundamentally overcome the intrinsic thermomechanical limitations of commercial epoxy matrices and often exacerbates rheological issues and processability deterioration at ultra-high filler loadings [25,26]. The second category is matrix modification by incorporating flexible modifiers, such as liquid rubbers, silicone oligomers, and organic toughening segments, to alleviate stress concentration and enhance the fracture toughness of cured epoxy systems [27,28,29]. Unfortunately, these modifiers typically cause a significant decrease in glass transition temperature (T_g_), impaired thermomechanical stability, and even undesirable phase separation in the crosslinked network [30,31,32]. Therefore, there is an urgent need for a matrix-level modification strategy that can simultaneously improve the thermal resistance of epoxy resins without severely compromising their intrinsic processability.

Reactive mesogenic epoxy monomers, also known as liquid–crystalline epoxies, have attracted increasing research attention for the modification of high-performance thermosets in electronic packaging. This is attributed to their unique molecular structure, which integrates rigid aromatic mesogenic moieties with flexible aliphatic spacer segments. The rigid mesogenic units enhance the chain regularity and intermolecular interactions of the cured resin, thereby effectively increasing its T_g_ and thermal stability. Meanwhile, the flexible spacer segments relieve local stress concentration in densely crosslinked networks and improve the fracture energy dissipation capability of the material [33,34]. Furthermore, reactive LCE monomers can directly participate in the crosslinking reaction of the epoxy system, and their structure–property relationships are strongly governed by the curing protocol and molecular assembly behavior of the system [35,36].

Existing studies have confirmed that mesogenic units can improve the molecular ordering of the resin matrix and, in some cases, enhance the intrinsic phonon transport efficiency to boost thermal conductivity. For instance, Hong and Goh [37] systematically reviewed the research progress of liquid–crystalline epoxies for high-thermal-conductivity applications. Dang et al. [38] elevated the intrinsic thermal conductivity of epoxy systems via the introduction of a biphenyl mesogen-containing co-curing agent. Yuan et al. [39] enhanced heat transport in liquid–crystalline epoxy systems through a rationally designed interlocked polymer network strategy. Nevertheless, most previous studies have focused on neat liquid–crystalline epoxy matrices or lightly filled model epoxy systems. In contrast, highly filled commercial thermally conductive potting compounds are more complex, because their final performance is governed not only by the intrinsic structure of the epoxy matrix, but also by filler packing, filler–matrix interfacial interactions, curing behavior, rheological processability, and mechanical integrity. Consequently, although reactive mesogenic monomers can regulate the matrix structure [40], it remains unclear whether they can improve the thermomechanical property balance of practical highly filled potting systems while preserving the continuity of the thermally conductive filler network. To address this question, the present work incorporates a mesogen-containing reactive epoxy monomer into a highly filled commercial thermally conductive potting compound via a dual-curing strategy and evaluates its effects on curing behavior, thermomechanical properties, rheological processability, mechanical performance, and thermal conductivity.

In this study, a mesogen-containing reactive epoxy monomer (LCE) was designed and synthesized and then incorporated into a commercial thermally conductive epoxy potting compound. The structure of LCE was characterized via Fourier-transform infrared spectroscopy (FT-IR) and proton nuclear magnetic resonance (^1^H NMR). Differential scanning calorimetry (DSC), polarized optical microscopy (POM), and curing analysis were further used to clarify its liquid–crystalline behavior and curing mode in the potting compound system. The effects of LCE loading on the glass transition behavior, thermal stability, thermal conductivity, room-temperature rheological properties, tensile performance, and fracture-surface morphology of the cured potting compounds were systematically investigated. The aim of this study is to clarify whether the introduction of LCE can effectively enhance the thermal resistance of commercial thermally conductive epoxy potting compounds while maintaining a balance among processability, tensile performance, and heat-transfer capability.

## 2. Materials and Methods

### 2.1. Materials

3,3′,5,5′-Tetramethylbiphenyl-4,4′-diol (C_16_H_18_O_2_, AR), p-hydroxybenzoic acid (C_7_H_6_O_3_, AR), p-toluenesulfonic acid (C_7_H_8_O_3_S, AR), 6-bromo-1-hexene (C_6_H_11_Br, AR), 18-crown-6 (C_12_H_24_O_6_, AR), and m-chloroperoxybenzoic acid (m-CPBA, C_7_H_5_ClO_3_, AR) were purchased from Macklin Biochemical Co., Ltd. (Shanghai, China). Anhydrous potassium carbonate (K_2_CO_3_, AR) was obtained from Xilong Chemical Plant (Shantou, China). p-xylene (C_8_H_10_, AR) and dichloromethane (CH_2_C_l2_, AR) were supplied by Tianjin Damao Chemical Reagent Factory (Tianjin, China). 4,4′-Diaminodiphenylmethane (DDM, C_13_H_14_N_2_, AR) was purchased from Aladdin Biochemical Technology Co., Ltd. (Shanghai, China). N,N-Dimethylformamide (DMF, C_3_H_7_NO, AR) was purchased from Tianjin Fuyu Chemical Co., Ltd. (Tianjin, China). Sodium sulfite (Na_2_SO_3_, AR), sodium chloride (NaCl, AR), and sodium bicarbonate (NaHCO_3_, AR) were provided by Tianjin Jindong Tianzheng Fine Chemical Reagent Factory and Tianjin Tianda Chemical Reagent Factory (Tianjin, China). The commercial thermally conductive epoxy potting base resin and its matched proprietary curing agent were supplied by Elantas Electrical Insulation Materials (Zhuhai) Co., Ltd. (Zhuhai, China).

### 2.2. Characterization

FT-IR spectra were collected on a NICOLET iS50 FT-IR spectrometer (Thermo Fisher Scientific Co., Ltd., Waltham, MA, USA) over a wavenumber range of 500–4000 cm^−1^.

^1^H NMR spectra were recorded on a Bruker AVANCE NEO 600 MHz spectrometer (Bruker Co., Ltd., Bremen, Germany) at 25 °C, using CDCl_3_ or DMSO-d_6_ as the solvent.

The phase transition behavior of LCE was characterized using a Leica DM4P POM (Leica Microsystems Co., Ltd., Wetzlar, Germany) equipped with a Linkam THMS 600 hot stage (Linkam Scientific Instruments Co., Ltd., Redhill, UK) at a heating rate of 10 K min^−1^.

DSC measurements were performed using a Q20 calorimeter (TA Instruments Co., Ltd., New Castle, DE, USA) under a nitrogen atmosphere. The thermal-transition behavior of LCE and the cured potting compounds was investigated from 30 to 200 °C at 10 K min^−1^. Non-isothermal curing kinetics of the isolated LCE/DDM subsystem were analyzed at heating rates of 5, 10, 15, and 20 K min^−1^ using the Starink isoconversional method. To qualitatively assess residual curing behavior, representative uncured and cured formulations containing 0%, 10%, 30%, and 50% LCE were scanned from 30 to 250 °C at 10 K min^−1^. The cured samples were prepared using the same curing procedure as the final potting compounds.

Thermogravimetric analysis (TGA) and derivative thermogravimetry (DTG) tests were carried out on a Q500 analyzer (TA Instruments Co., Ltd., New Castle, DE, USA) under a nitrogen atmosphere from 30 to 800 °C at a heating rate of 20 K min^−1^.

Dynamic mechanical analysis (DMA) was conducted using a Q800 instrument (TA Instruments Co., Ltd., New Castle, DE, USA) in tensile mode at a frequency of 1 Hz and a strain amplitude of 0.1%. The temperature was increased from 30 to 200 °C at a heating rate of 5 K min^−1^.

Room-temperature rheological properties were measured using a HAAKE rotational rheometer (Thermo Fisher Scientific Co., Ltd., Dreieich, Germany) equipped with a 25 mm parallel-plate geometry and a fixed gap of 1.0 mm. Before measurement, each uncured potting compound was allowed to rest for approximately 5 min at 25 °C to reduce the influence of loading history. Time-dependent complex viscosity was subsequently recorded at 25 °C using an oscillatory frequency of 1 Hz and a strain amplitude of 1.0%. The temperature of 25 °C was selected to represent practical room-temperature processing conditions for potting applications. Measurements were continued until the complex viscosity exceeded approximately 120 Pa·s.

The thermal conductivity of the cured samples was measured using a Hot Disk TPS 2500S thermal constants analyzer (Hot Disk AB, Gothenburg, Sweden) at 25 °C. A single measurement was performed for each formulation under identical testing conditions.

Tensile properties were tested on an Instron 5567A universal testing machine (Instron Co., Ltd., Norwood, MA, USA) at a crosshead speed of 5 mm/min, using film specimens with dimensions of 6 mm × 40 mm and a thickness of 40–60 μm.

The cryo-fractured cross-sectional morphology was examined using an SU8220 field-emission scanning electron microscope (Hitachi High-Technologies Corporation, Tokyo, Japan) after sputter-coating with gold.

SEM–EDS elemental mapping was performed separately on representative fractured surfaces of the 0%, 10%, 30%, and 50% LCE samples using an SU8600 ultra-high-resolution field-emission scanning electron microscope (Hitachi High-Tech Corporation, Tokyo, Japan). The distributions of C, O, and Al were recorded to provide qualitative information on local elemental variation.

### 2.3. Synthesis of the Liquid–Crystalline Epoxy Monomer LCE

#### 2.3.1. Synthesis of Compound **1**

**Figure 1:**
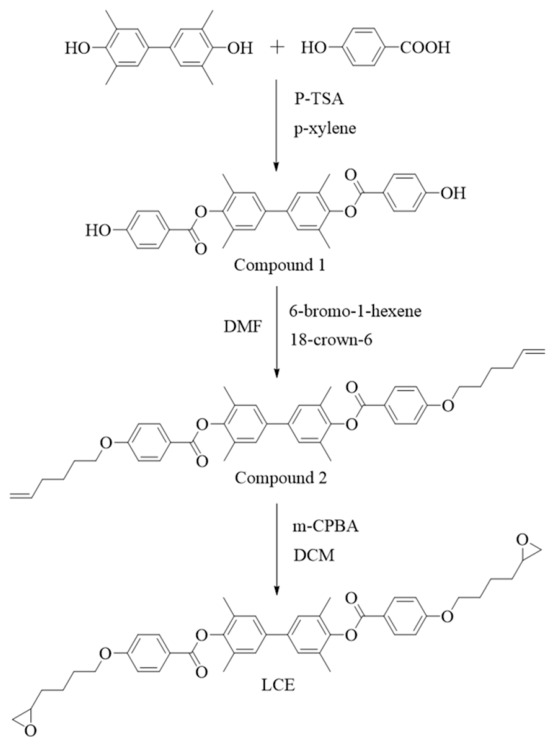
Synthetic route of the reactive epoxy monomer LCE.

Compound **1** was synthesized through an esterification reaction. 3,3′,5,5′-Tetramethylbiphenyl-4,4′-diol (2.42 g, 10 mmol), p-hydroxybenzoic acid (2.76 g, 20 mmol), p-toluenesulfonic acid (0.17 g, 1 mmol), and p-xylene (100 mL) were placed in a 250 mL three-necked flask fitted with a Dean-Stark trap and a reflux condenser. The suspension was refluxed under mechanical stirring for 24 h, and the water generated during esterification was removed continuously through the trap. After cooling to room temperature, the precipitated product was separated by filtration, washed three to four times with ethanol, and dried under vacuum at 60 °C for 12 h. A white solid was obtained as Compound **1** (Figure 1), with an isolated yield of 78%.

#### 2.3.2. Synthesis of Compound **2**

Compound **2** was prepared via etherification of Compound **1**. Compound **1** (4.82 g, 10 mmol), anhydrous K_2_CO_3_ (6.91 g, 50 mmol), 18-crown-6 (0.26 g, 1 mmol), and DMF (100 mL) were introduced into a three-necked flask. 6-Bromo-1-hexene (4.89 g, 30 mmol) was then added dropwise while stirring was maintained. The reaction mixture was kept at 60 °C for 24 h. After the reaction, the mixture was slowly poured into excess deionized water to precipitate the product. The solid was collected by filtration, washed two to three times with absolute ethanol, and dried in a vacuum oven at 60 °C for 12 h. Compound **2** (Figure 1) was obtained as a white solid in 90% yield.

#### 2.3.3. Synthesis of LCE

LCE was obtained via epoxidation of the terminal double bonds in Compound **2**. Compound **2** (6.47 g, 10 mmol) was dissolved in dichloromethane (50 mL) in a three-necked flask. The solution was cooled in an ice-water bath, and m-CPBA (8.63 g, 50 mmol) was added portionwise under vigorous stirring. The reaction mixture was then warmed to 30 °C and stirred for 24 h. The insoluble m-CPBA by-product was removed by filtration. The filtrate was washed sequentially with saturated sodium sulfite solution, saturated sodium bicarbonate solution, and saturated sodium chloride solution, followed by drying over anhydrous sodium sulfate. After filtration and solvent removal under reduced pressure, the crude product was recrystallized from toluene and vacuum-dried to give LCE (Figure 1) as an orange solid. The isolated yield was 69%.

### 2.4. Preparation of LCE-Modified Potting Compounds

The LCE-modified potting compounds were prepared according to the formulations listed in Table 1. The commercial thermally conductive epoxy potting base resin was fixed at 100 parts by mass, and the matched proprietary curing agent was added at a constant base-resin-to-curing-agent mass ratio of 100:11. LCE was incorporated at 0, 10, 20, 30, 40, and 50 parts by mass relative to 100 parts of the base resin. DDM was employed as the curing agent for LCE and was added at a stoichiometric epoxy-to-amine equivalent ratio, corresponding to an LCE/DDM molar ratio of 2:1. For preparation, LCE was first dispersed in the base resin at room temperature, followed by the addition of DDM and mixing until no visible nonuniformity was observed. The proprietary curing agent was then added, and the formulation was stirred for 5–10 min. After vacuum deaeration, the mixtures were cast into molds and cured at 25 °C for 24 h, 50 °C for 12 h, and 175 °C for 4 h. The cured samples were designated as 0%, 10%, 20%, 30%, 40%, and 50% LCE. These labels are nominal formulation identifiers representing the amount of LCE added relative to 100 parts by mass of the base resin rather than the actual LCE content in the complete formulation. The actual LCE mass fractions in the complete formulations are reported separately in Table 1.

## 3. Results and Discussion

### 3.1. Structure of LCE and Intermediates

**Figure 2:**
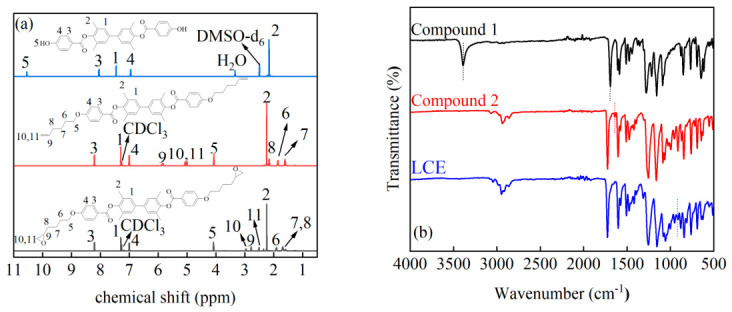
(**a**) ^1^H NMR spectra of Compound **1**, Compound **2**, and LCE; (**b**) FT-IR spectra of Compound **1**, Compound **2**, and LCE.

The chemical structures of LCE and its intermediates were characterized via FT-IR and ^1^H NMR spectroscopy. As shown in Figure 2a, Compound **1** exhibited a characteristic phenolic hydroxyl proton signal at δ 10.55 ppm, indicating the successful formation of Compound **1**. After etherification, this signal disappeared, while a characteristic methylene signal linked to the ether oxygen emerged at δ 4.08 ppm. In addition, signals associated with terminal double bonds appeared in the range of δ 4.9–5.9 ppm, indicating that the terminal alkenyl segments had been successfully grafted onto the molecule, confirming the formation of Compound **2**. After epoxidation, the original alkene signals completely disappeared, and new epoxy-related signals appeared in the range of δ 2.7–3.4 ppm, demonstrating the successful formation of the target terminal epoxy structure of LCE.

FT-IR spectroscopy was further used to confirm the structural evolution of LCE, as shown in Figure 2b. Compound **1** exhibited a broad absorption band near 3400 cm^−1^, which can be assigned to the O-H stretching vibration, and a strong absorption band near 1700 cm^−1^ corresponding to the C=O stretching vibration of the ester group, indicating the successful formation of the aromatic ester structure. Compared with Compound **1**, the hydroxyl absorption near 3400 cm^−1^ completely disappeared in the spectrum of Compound **2**, confirming the full consumption of phenolic hydroxyl groups during etherification. Concurrently, a new absorption appeared near 3000–3100 cm^−1^, which can be assigned to the =C-H stretching vibration of the terminal alkenyl group, verifying the successful introduction of the unsaturated side chains. After epoxidation, the alkene-related absorptions were significantly attenuated, and new characteristic absorptions associated with the oxirane (epoxy) ring appeared at 918 cm^−1^. These combined FT-IR and ^1^H NMR results unambiguously confirm the successful synthesis of the target liquid–crystalline epoxy monomer, LCE.

### 3.2. Phase Transition and Liquid–Crystalline Behavior of LCE

**Figure 3:**
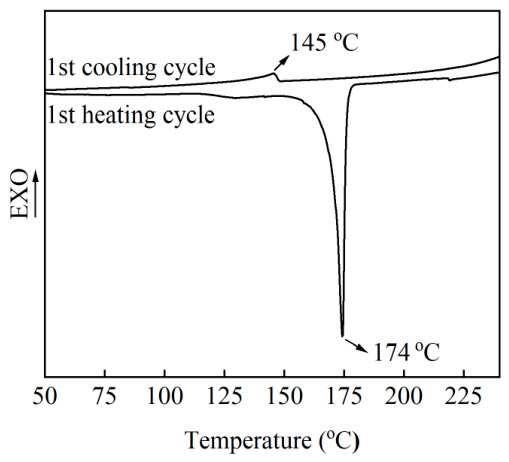
DSC curves for the first heating and first cooling cycles of the LCE monomer.

The phase-transition behavior and liquid–crystalline characteristics of the synthesized LCE were investigated via DSC and POM. As shown in Figure 3, the LCE monomer exhibited a sharp endothermic transition near 174 °C during the first heating cycle. Combined with the POM observations, this transition may be associated with melting into a liquid–crystalline mesophase. The absence of a separately resolved clearing peak in the DSC curve suggests that the mesophase exists over a relatively narrow temperature interval. During cooling, an exothermic transition was observed near 145 °C, indicating the reverse phase transition.

**Figure 4:**
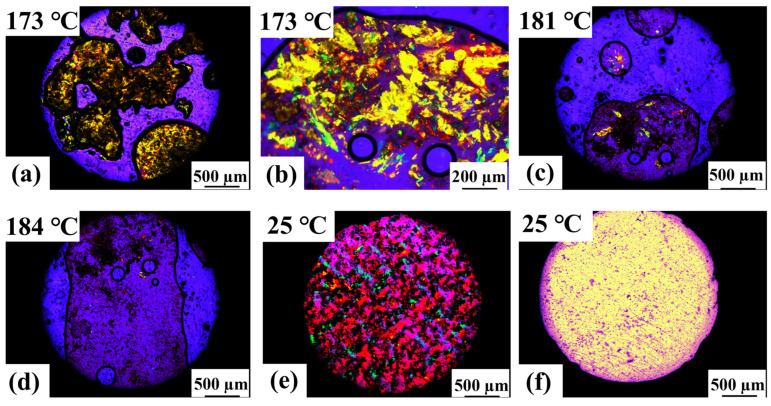
POM images of the LCE monomer and cured systems under different conditions: (**a**) LCE monomer at 173 °C; (**b**) enlarged POM image of the LCE monomer at 173 °C; (**c**) LCE monomer at 181 °C; (**d**) LCE monomer at 184 °C; (**e**) LCE/DDM system cured at 175 °C and observed at 25 °C; (**f**) dual-curing potting compound cured according to the programmed curing procedure and observed at 25 °C.

The POM images in Figure 4a–d further demonstrate the temperature-dependent optical anisotropy of LCE. Distinct birefringent textures were observed at 173 °C, whereas the birefringence progressively weakened at 181 and 184 °C, indicating a gradual transition toward the isotropic state at higher temperatures. The behavior of LCE in the cured systems was also examined. The cured LCE/DDM model system exhibited birefringent features at 25 °C after curing at 175 °C (Figure 4e), which may be associated with locally retained anisotropic regions. In contrast, no clearly discernible birefringent texture was observed via POM in the dual-cured potting compound after the programmed curing schedule (Figure 4f). This difference may result from dilution by the commercial matrix and the spatial constraints imposed by the inorganic fillers and multicomponent crosslinked network. Therefore, although the LCE monomer exhibits liquid–crystalline behavior, no macroscopic retention of liquid–crystalline order was detected via POM in the highly filled cured potting compound. The subsequent property changes may instead be associated with the incorporation of rigid mesogenic units and the resulting changes in segmental mobility, network structure, and rheological behavior, rather than with retained long-range liquid–crystalline order [41].

### 3.3. Dual-Curing Design and Curing Behavior

**Figure 5:**
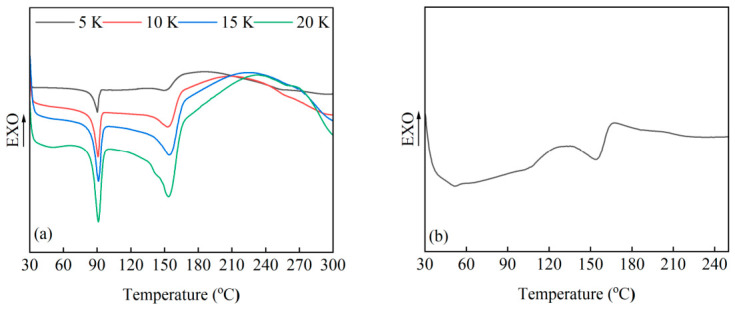
DSC curves illustrating the reactivity difference: (**a**) non-isothermal curing behavior of the LCE/DDM system at different heating rates; (**b**) the LCE/proprietary curing-agent system at 20 K min^−1^.

To determine whether LCE could effectively participate in the crosslinking reaction of the matrix, its curing behavior was first evaluated. Figure 5b shows the non-isothermal DSC curve of the LCE/proprietary curing agent system at a heating rate of 20 K min^−1^. No distinct curing exothermic peak was observed; instead, only a broad and weak exothermic signal appeared between 120 and 180 °C. This result indicates that the proprietary curing agent supplied with the commercial potting compound has limited reactivity toward LCE under the experimental conditions. If this curing pathway alone were used, the effective incorporation of LCE into the crosslinked network might be limited, which could compromise network integrity and the final performance of the materials.

To address this limitation, DDM was introduced as a co-curing agent to establish a dual-curing system composed of the commercial potting base resin/proprietary curing agent pair and the LCE/DDM subsystem. As shown in Figure 5a, the LCE/DDM system displayed a monomer melting endothermic peak near 90 °C followed by a clear curing exotherm in the range of 150–240 °C, indicating that DDM effectively promoted the curing and crosslinking of LCE. The regular shift of the exothermic peak toward higher temperatures with increasing heating rate is consistent with the typical behavior of a non-isothermal curing process.

To further evaluate the curing characteristics of the LCE/DDM subsystem, non-isothermal curing kinetics were analyzed using the Starink isoconversional method based on DSC measurements conducted at heating rates of 5, 10, 15, and 20 K min^−1^. The corresponding results are presented in Figure S1 and Table S1. Over the conversion range of α = 0.1–0.9, the apparent activation energy decreased from 87.90 to 24.86 kJ mol^−1^, with an average value of 46.06 kJ mol^−1^. The variation in apparent activation energy indicates that the effective reaction barrier changes as curing proceeds. Because the melting transition partially overlapped with the broad curing thermal event, these values are treated as approximate kinetic descriptors of the isolated LCE/DDM subsystem. Nevertheless, the results provide supplementary evidence of a temperature-dependent reaction between LCE and DDM and support the feasibility of the proposed dual-curing pathway. Importantly, these kinetic parameters are specific to the subsystem and are not intended to quantify cure completion of the fully formulated potting compounds.

### 3.4. Qualitative Assessment of Residual Curing Behavior by DSC

**Figure 6:**
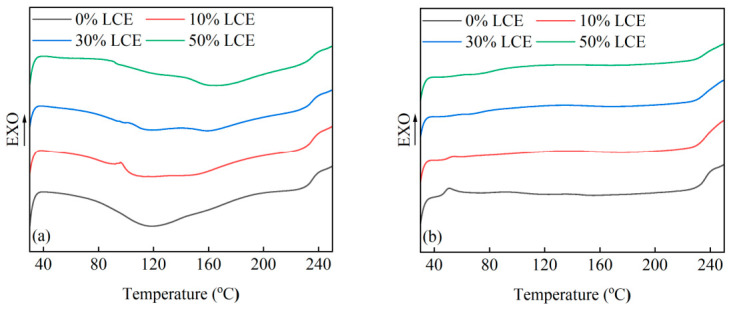
DSC thermograms of formulations containing different LCE contents: (**a**) uncured formulations; (**b**) cured formulations after the programmed curing schedule.

To further evaluate the residual curing behavior of the dual-curing system, DSC measurements were performed on representative uncured and cured formulations containing 0%, 10%, 30%, and 50% LCE. As shown in Figure 6a, the uncured formulations exhibited broad and overlapping thermal events between approximately 90 and 220 °C, reflecting contributions from thermal transitions and curing reactions. Following the programmed curing process, these thermal features were substantially reduced, and no distinct residual exothermic peak that could be reliably separated from the baseline was detected in the cured formulations (Figure 6b). Because the remaining baseline fluctuations prevented objective selection of the integration limits, the residual reaction enthalpy was not calculated. The post-cure DSC results were therefore used for qualitative assessment of detectable residual curing behavior rather than quantitative determination of the degree of cure. Although Raman and near-infrared spectroscopy can provide more direct information on functional-group conversion during curing processes [42], post-cure DSC was employed as a practical complementary method for the present highly filled commercial system. The DSC results were not interpreted as direct spectroscopic quantification of epoxy conversion.

### 3.5. Thermal Properties of Modified Potting Compounds

**Figure 7:**
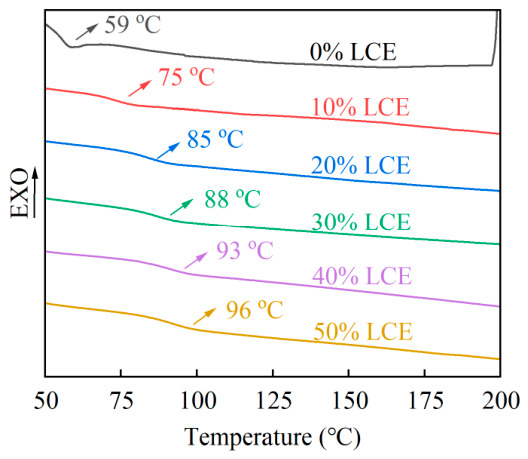
DSC curves of potting compound samples with different LCE contents.

The thermal behavior of the modified potting compounds was evaluated via DSC and TGA. Figure 7 shows that T_g_ increased steadily with increasing LCE loading. The unmodified matrix exhibited a T_g_ of 59 °C, whereas the samples denoted as 10% LCE, 20% LCE, 30% LCE, 40% LCE, and 50% LCE exhibited T_g_ values of 75, 85, 88, 93, and 96 °C, respectively. These results indicated that the introduction of LCE increasingly restricted segmental motion within the cured network. This behavior is associated with the rigid aromatic mesogenic structure of LCE, which improves local network regularity and strengthens intermolecular interactions.

**Figure 8:**
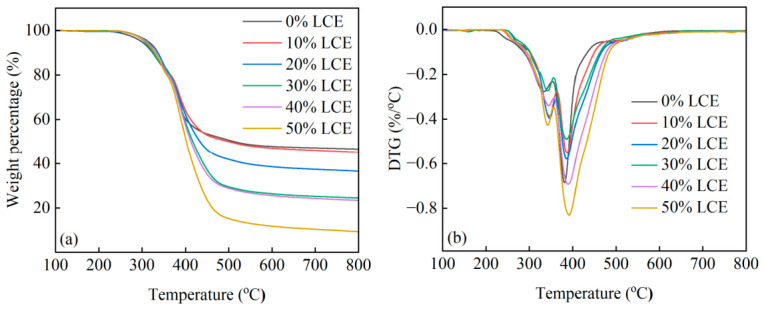
(**a**) TGA curves of potting compound samples with different LCE loadings; (**b**) DTG curves of potting compound samples with different LCE loadings.

The thermal stability of the modified systems was evaluated via TGA. The corresponding TGA and DTG curves are shown in Figure 8, and the characteristic parameters are summarized in Table 2. For the unmodified sample, the 5% mass-loss temperature (T_d,5%_), 10% mass-loss temperature (T_d,10%_), and maximum decomposition temperature (T_d,max_) were 298, 324, and 382 °C, respectively. After LCE incorporation, T_d,5%_ ranged from 306 to 314 °C, while T_d,10%_ varied between 326 and 337 °C, showing no clear monotonic dependence on LCE content. These variations suggest that the initial degradation behavior is sensitive to differences in formulation composition, local network structure, and possible low-molecular-weight components.

In contrast, T_d,max_ remained within 384–393 °C for the LCE-containing samples and within 382–393 °C across all formulations, suggesting that the principal thermal decomposition process of the epoxy network was largely preserved after LCE incorporation. However, the residual mass at 800 °C decreased from 47% for the unmodified sample to 9% for the 50% LCE sample. This decrease should not be interpreted solely as a loss of intrinsic thermal stability. The commercial potting compound contains a high fraction of thermally stable inorganic fillers, and the addition of increasing amounts of LCE and DDM reduces their relative mass fraction in the final formulations. This filler-dilution effect is considered the primary reason for the lower residual mass. Changes in the organic network structure and char-forming behavior may also contribute, although these effects cannot be distinguished from the present TGA data without further residue-composition or evolved-gas analysis. Therefore, the thermal stability of the modified systems should be evaluated by considering T_d,5%_, T_d,10%_, T_d,max_, and residual mass together. Overall, LCE incorporation did not substantially alter the main decomposition temperature, whereas high LCE loadings reduced the final residue primarily because of the lower relative inorganic-filler content.

### 3.6. Dynamic Mechanical Properties

**Figure 9:**
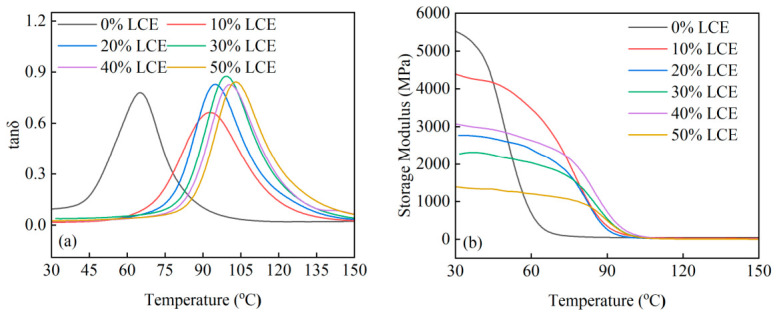
(**a**) Temperature-dependent tan δ curves of potting compound samples with different LCE loadings; (**b**) temperature-dependent storage modulus curves of potting compound samples with different LCE loadings.

DMA was conducted on film specimens of the LCE-modified potting compounds, and the results are summarized in Figure 9 and Table 3. The tan δ peak temperature (T_g_) progressively increased from 65 °C for the 0% LCE sample to 103 °C for the 50% LCE sample, indicating increasingly restricted segmental mobility within the crosslinked network. Regarding the storage modulus (E′), the 10% LCE sample exhibited the highest glassy modulus (4016 MPa at 50 °C). With further LCE addition, E′ decreased for the 20% and 30% LCE samples, rebounded at 40% LCE, and dropped markedly at 50% LCE. This non-monotonic variation may result from competing structural effects. The incorporation of bulky mesogenic monomers may reduce the compactness of the original crosslinked network, whereas the rigid mesogenic segments may provide local physical reinforcement. The rebound observed at 40% LCE may therefore reflect a temporary balance between reduced network compactness and the reinforcing contribution of the rigid mesogenic segments. Because direct structural evidence of mesogen ordering was not obtained, this interpretation remains tentative. At 50% LCE, increased microstructural heterogeneity and reduced network integrity may contribute to the marked decrease in E′.

Although the system contains multiple components, all samples exhibited a single dominant tan δ peak within the investigated temperature range. This suggests that no clearly resolved additional relaxation process attributable to a distinct phase was detected within the sensitivity of the DMA measurement. However, a single tan δ peak alone is insufficient to demonstrate complete molecular-level miscibility or network homogeneity. The FWHM values remained similar for the 0–20% LCE samples (16.5–16.9 °C) and increased to 20.1–24.0 °C for the 30–50% LCE samples. This broadening is consistent with a wider distribution of segmental relaxation times and greater local structural heterogeneity at higher LCE contents. Consequently, the DMA results indicate the absence of clearly resolved additional relaxation processes rather than definitive proof of complete molecular-level miscibility. The microstructural characteristics of representative cured samples are further examined via SEM and SEM–EDS mapping in Section 3.9.

### 3.7. Room-Temperature Rheological Behavior

**Figure 10:**
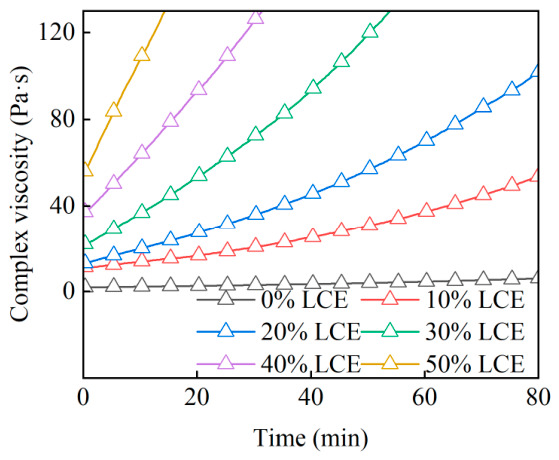
Complex viscosity–time curves of potting compound samples with different LCE contents at 25 °C.

While LCE incorporation modifies the post-cure thermomechanical properties of the potting compounds, its influence on room-temperature processability must also be considered. Because all samples were evaluated under identical oscillatory conditions, the viscosity evolution provides a comparative assessment of formulations with different LCE contents. The test temperature of 25 °C was selected to represent practical mixing and dispensing conditions. Rheological measurements were performed using a 25 mm parallel-plate geometry with a fixed gap of 1.0 mm at a frequency of 1 Hz and a strain amplitude of 1.0%. These conditions were kept constant for all formulations to permit comparison while limiting disturbance of the highly filled systems. The corresponding storage modulus (G′) and loss modulus (G″) curves are provided in Figure S2. Figure 10 shows the time-dependent evolution of complex viscosity (η*) at 25 °C. The initial η* increased from 1.96 Pa·s for the 0% LCE sample to 56.17 Pa·s for the 50% LCE sample, indicating increased resistance to flow with increasing LCE content. Viscosity buildup also became more pronounced at higher LCE loadings. The 50%, 40%, and 30% LCE samples reached the selected viscosity threshold of 100 Pa·s after approximately 8.72, 22.68, and 43.02 min, respectively. Thus, increasing LCE content progressively shortened the time available before this threshold was reached. In contrast, the 10% and 20% LCE formulations required longer times to reach the selected viscosity threshold.

The rheological evolution of highly filled thermosetting systems may reflect both physical interactions and early-stage network development. Previous rheological and spectroscopic studies have shown that viscosity can be influenced by filler-induced interactions and the advancement of curing reactions prior to gelation [43,44,45]. Therefore, the viscosity growth observed here likely results from the combined effects of LCE content, filler–matrix interactions, and early-stage network evolution. These results should be interpreted as a comparative assessment of room-temperature viscosity evolution and processability within the monitored time window, rather than as a direct measurement of complete curing behavior at elevated temperatures. Thus, although LCE improved certain thermomechanical properties, it also increased viscosity and reduced the available processing window. Similar trade-offs between processability and cured properties have been reported for other modified epoxy systems [46].

### 3.8. Tensile Properties

**Figure 11:**
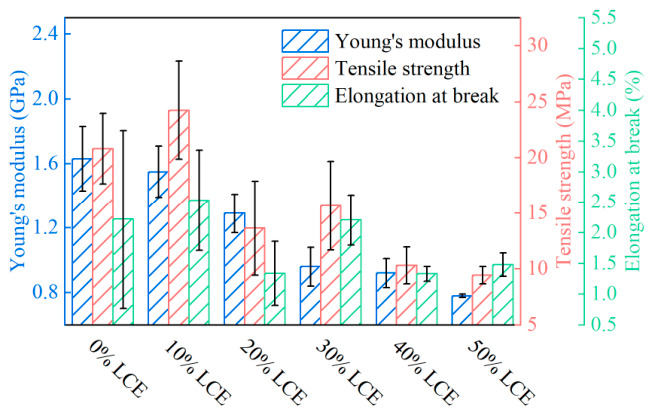
Tensile properties of potting compound samples with different LCE contents.

It should be noted that the tensile tests were performed on thin-film specimens with a thickness of 40–60 μm. This geometry was selected to reduce the influence of thickness-dependent curing gradients and bulk casting defects, thereby enabling a consistent comparison of the material-level mechanical response of different formulations under identical specimen geometry and curing conditions. Therefore, the tensile results should not be interpreted as a direct representation of the mechanical behavior of bulk potting components under service conditions. Further evaluation using bulk or application-scale specimens would be required to assess practical mechanical reliability. The tensile properties of the modified potting compounds exhibited a non-monotonic dependence on LCE content (Figure 11). The unmodified sample showed a tensile strength of 20.82 MPa, an elongation at break of 2.22%, and a Young’s modulus of 1.63 GPa. At 10% LCE, the tensile strength increased to 24.26 MPa and the elongation at break reached 2.53%, indicating an improved balance between strength and elongation at low LCE content.

With further increases in LCE content, the tensile strength gradually decreased. The 30% LCE sample retained an elongation at break of 2.21%, whereas the tensile strengths of the 40% and 50% LCE samples decreased markedly to 10.33 and 9.45 MPa, respectively. Meanwhile, Young’s modulus decreased from 1.63 GPa for the unmodified sample to 0.78 GPa for the 50% LCE sample, indicating a progressive reduction in stiffness. These results show that LCE does not provide continuous strengthening across the entire composition range. For the representative formulations examined via post-cure DSC, no distinct residual exothermic peak that could be reliably separated from the baseline was detected. This observation suggests that detectable residual curing behavior is unlikely to be the sole factor governing the mechanical trend. The observed differences may also be associated with variations in network structure, filler–matrix interactions, and microstructural uniformity. At low LCE contents, the mesogenic segments may improve stress transfer within the crosslinked network. At high LCE contents, the associated increase in viscosity may hinder filler redistribution and interfacial wetting during processing and curing, leading to reduced microstructural uniformity and mechanical performance. This interpretation is consistent with previous studies showing that the mechanical response of modified epoxy systems is strongly influenced by network architecture, microstructural uniformity, and local deformation mechanisms [47]. Fracture-surface morphology and representative SEM–EDS results are discussed in the following section to further examine the relationship between microstructure and tensile performance.

### 3.9. Fracture-Surface Morphology

**Figure 12:**
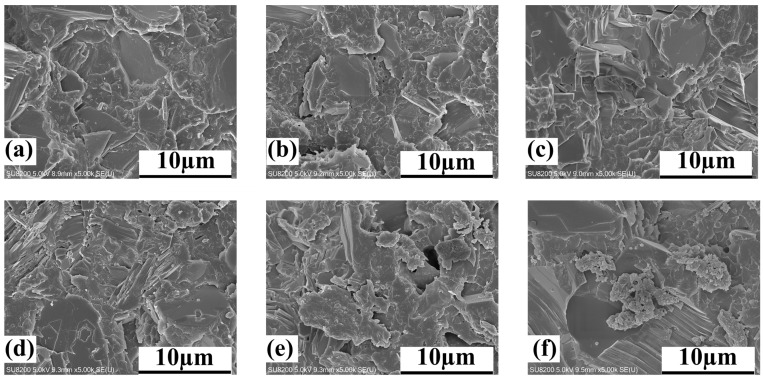
SEM images of cryo-fractured cross-sectional surfaces of potting compound samples with different LCE contents: (**a**) 0% LCE; (**b**) 10% LCE; (**c**) 20% LCE; (**d**) 30% LCE; (**e**) 40% LCE; (**f**) 50% LCE.

To further examine the microstructural features potentially associated with the non-monotonic tensile properties, the cryo-fractured cross-sectional surfaces of the cured potting compounds were observed via SEM, as shown in Figure 12. The 0% LCE sample exhibited a relatively smooth and featureless surface, consistent with brittle fracture characteristics. In comparison, the 10% LCE sample showed a rougher surface containing tear ridges, wrinkles, and local crack deflection. These features are consistent with a more tortuous fracture path and with the increased tensile strength and elongation at break of this formulation.

The 20% and 30% LCE samples also exhibited rough surfaces with lamellar peeling, step-like features, and local crack deflection. Post-cure DSC analysis of representative formulations revealed no distinct residual exothermic peak that could be reliably separated from the baseline. Therefore, detectable residual curing behavior is unlikely to be the sole factor governing the observed mechanical trend. Variations in network structure, filler–matrix interactions, and microstructural uniformity may also contribute. At higher LCE loadings, morphological nonuniformity became more apparent. Figure 12e,f shows exposed filler surfaces, interfacial defects, and locally nonuniform regions in the 40% and 50% LCE samples. Excessive LCE incorporation and the associated increase in viscosity may hinder filler redistribution and effective resin wetting of the fillers during processing and curing. These features may act as stress-concentration sites and contribute to the reduction in tensile strength and stiffness at high LCE contents. Similar effects of filler aggregation and interfacial defects on the mechanical performance of highly filled composites have been reported previously [48].

To further assess filler distribution and local elemental variation, SEM–EDS elemental mapping was performed separately on representative fractured surfaces of the 0%, 10%, 30%, and 50% LCE samples, as shown in Figures S3 and S4. The distributions of C, O, and Al provide qualitative information on the local filler–matrix arrangement at the observed length scale. The 0%, 10%, and 30% LCE samples exhibited comparatively uniform elemental distributions, whereas the 50% LCE sample showed greater local variation, including C-rich regions and locally concentrated Al/O signals. These observations indicate greater local compositional variation in the 50% LCE sample than in the selected lower-LCE formulations. However, the SEM–EDS results are not interpreted as quantitative evidence of phase composition or molecular-level miscibility.

### 3.10. Thermal Conductivity

**Figure 13:**
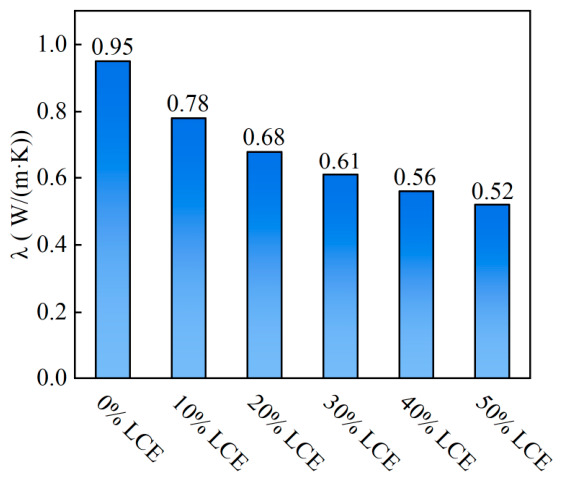
Thermal conductivity of potting compound samples with different LCE contents at 25 °C. Each value represents a single measurement.

The single-point thermal-conductivity measurements showed a monotonic decreasing trend in the measured values with increasing LCE content. As shown in Figure 13, the measured value decreased from 0.95 W/(m·K) for the unmodified sample to 0.78, 0.68, 0.61, 0.56, and 0.52 W/(m·K) for the 10%, 20%, 30%, 40%, and 50% LCE samples, respectively. These results suggest that LCE incorporation did not improve heat transfer in the present highly filled potting system. Two factors may contribute to this observed trend. First, the increased viscosity at high LCE loadings may hinder filler rearrangement and reduce effective filler–filler contact during processing and curing, thereby impeding the formation of continuous thermally conductive pathways. Second, the exposed filler surfaces, interfacial defects, and locally nonuniform regions observed in Figure 12f may be associated with less effective resin wetting at high LCE loading. These features may increase interfacial thermal resistance and phonon scattering, thereby reducing heat-transfer efficiency. Increasing LCE content may also introduce greater local structural heterogeneity and further disrupt the continuity of thermally conductive pathways. This interpretation is consistent with previous studies showing that thermal transport in epoxy composites is influenced by filler–matrix and filler–filler interfacial thermal resistance [49]. Nevertheless, this proposed mechanism remains qualitative because no quantitative interface model was established.

From a practical potting perspective, high LCE loading may introduce both processing and heat-dissipation challenges. The simultaneous increase in room-temperature viscosity and apparent decrease in thermal conductivity may hinder mixing, dispensing, gap filling, and bubble release during processing while reducing heat-transfer efficiency after curing. Each thermal-conductivity value reported here was obtained from a single measurement at 25 °C; therefore, statistical uncertainty and error bars are not provided. The results should be interpreted as preliminary comparative measurements obtained under identical testing conditions. Temperature-dependent and directional thermal-conductivity measurements were not performed. Although the samples were prepared via conventional casting without an external alignment field, possible local anisotropy cannot be excluded. Independent replicate, temperature-dependent, and directional measurements are required to confirm the observed trend and more fully evaluate the thermal-transport behavior of these materials.

## 4. Conclusions

A mesogen-containing reactive epoxy monomer (LCE) was successfully synthesized and incorporated into a commercial thermally conductive epoxy potting compound through a dual-curing strategy. DSC kinetic analysis of the isolated LCE/DDM subsystem indicated its curing reactivity, while post-cure DSC measurements of representative formulations showed no distinct residual exothermic peak that could be reliably separated from the baseline. LCE incorporation progressively increased the DMA-derived glass transition temperature (T_g_) from 65 °C to 103 °C, indicating increased restriction of segmental motion within the cured network. Meanwhile, the maximum decomposition temperature remained within 382–393 °C across all formulations, suggesting that the primary thermal decomposition behavior of the epoxy network was largely preserved. However, increasing LCE content also increased room-temperature viscosity and shortened the processing window. The initial complex viscosity increased from 1.96 Pa·s for the unmodified formulation to 56.17 Pa·s for the nominal 50% LCE formulation. In addition, the single-point thermal-conductivity measurements at 25 °C showed a monotonic decrease from 0.95 to 0.52 W/(m·K) with increasing LCE content, suggesting reduced heat-transfer capability and possible disruption of the thermally conductive network at higher LCE loadings. Mechanical testing further showed that low LCE contents improved tensile performance, whereas higher LCE contents resulted in reduced strength and stiffness, which may be associated with increased local microstructural heterogeneity.

Overall, reactive mesogenic modification provides a means of adjusting the thermomechanical properties of highly filled commercial thermally conductive potting compounds, although the accompanying trade-offs in processability and thermal transport must be considered. Among the investigated formulations, the nominal 10% LCE formulation provided the most balanced performance, with improved thermomechanical properties, a longer processing window than the higher-LCE formulations, and a relatively small decrease in the measured thermal-conductivity value. Practical potting behavior, long-term service reliability, replicate thermal-conductivity measurements, temperature-dependent thermal transport, and scalability require further investigation.

## Figures and Tables

**Table 1:** Formulations of LCE-modified thermally conductive potting compounds.

Sample	Potting Base Resin/Parts by Mass	Matched Curing Agent/Parts by Mass	LCE/Parts by Mass	DDM/Parts by Mass	Actual LCE Mass Fraction ^a^/wt%
0% LCE	100	11	0	0	0
10% LCE	100	11	10	1.5	8.2
20% LCE	100	11	20	2.9	14.9
30% LCE	100	11	30	4.4	20.6
40% LCE	100	11	40	5.8	25.5
50% LCE	100	11	50	7.3	29.7

All formulations are expressed in parts by mass. Sample labels (e.g., 10% LCE) are nominal formulation identifiers representing the amount of LCE added relative to 100 parts by mass of the potting base resin. ^a^ The actual LCE mass fraction was calculated as follows: $w_{LCE}\left(wt\%\right)=m_{LCE}/\left(m_{base\;resin}+m_{proprietary\;curing\;agent}+m_{LCE}+m_{DDM}\right)\times 100\%$.

**Table 2:** Thermal decomposition parameters of potting compound samples with different LCE loadings.

Sample	T_d,5%_ ^a^ (°C)	T_d,10%_ ^a^ (°C)	T_d,max_ ^b^ (°C)	C_y_ ^c^ (%)
0% LCE	298	324	382	47
10% LCE	314	337	385	45
20% LCE	314	337	384	37
30% LCE	306	326	387	25
40% LCE	307	330	392	23
50% LCE	310	332	393	9

^a^ Temperatures corresponding to 5% and 10% mass loss, obtained from TGA curves recorded under nitrogen from 30 to 800 °C at a heating rate of 20 K min^−1^; ^b^ Temperature at the maximum mass-loss rate, obtained from the DTG curves recorded under the same conditions; ^c^ Char residue, expressed as the remaining mass percentage at 800 °C.

**Table 3:** DMA characteristics of potting compound samples with different LCE loadings.

Sample	Storage Modulus E′ ^a^ (MPa)	tan δ Peak Temperature ^b^ (°C)	FWHM ^c^ (°C)
0% LCE	2765	65	16.5
10% LCE	4016	93	16.9
20% LCE	2604	95	16.6
30% LCE	2157	99	20.1
40% LCE	2826	101	22.8
50% LCE	1273	103	24.0

^a^ Storage modulus, E′, measured via DMA at 50 °C; ^b^ Temperature corresponding to the tan δ peak. ^c^ Full width at half maximum (FWHM) of the tan δ peak.

## Data Availability

The original contributions presented in this study are included in the article/Supplementary Materials. Further inquiries can be directed to the corresponding author.

## Supplementary Materials

The following supporting information can be downloaded at: https://www.mdpi.com/article/10.3390/polym18121503/s1, Figure S1: Curing kinetic analysis of the isolated LCE/DDM subsystem: (a) apparent activation energy (E_a_) as a function of conversion (α); (b) Starink plots of ln(β/T_α_1.92) versus 1/T_α_ at α = 0.1–0.9; Figure S2: Time-dependent storage modulus (G′) and loss modulus (G″) of the LCE-modified potting compounds measured at 25 °C, 1 Hz, and 1.0% strain: (a) 0% LCE, (b) 10% LCE, (c) 20% LCE, (d) 30% LCE, (e) 40% LCE, and (f) 50% LCE; Figure S3: SEM images and corresponding Al, C, O, and merged elemental maps of representative fractured surfaces of the 0% and 10% LCE samples; Figure S4: SEM images and corresponding Al, C, O, and merged elemental maps of representative fractured surfaces of the 30% and 50% LCE samples; Table S1: Starink kinetic parameters of the LCE/DDM subsystem at different degrees of conversion.
