# Supplementary material for: Mesogen-Containing Reactive Epoxy Monomer for Tuning the Thermal, Rheological, and Mechanical Properties and Fracture-Surface Morphology of Thermally Conductive Epoxy Potting Compounds
Source: Polymers (Basel). 2026 Jun 16;18(12):1503. doi: 10.3390/polym18121503 (PMC13306613; doi:10.3390/polym18121503)
Supplement: Supplementary file 1 [file polymers-18-01503-s001.zip › polymers-4349977-supplementary.pdf]

Supplementary Materials: Mesogen-Containing Reactive Epoxy Monomer for Tuning the Thermal, Rheological, and Mechanical Properties and Fracture-Surface Morphology of Thermally Conductive Epoxy Potting Compounds

***Section S1.*** *Curing Kinetic Analysis of the LCE/DDM Subsystem*

The non-isothermal curing kinetics of the isolated LCE/DDM subsystem were analyzed using the Starink isoconversional method. DSC measurements were conducted at heating rates (β) of 5, 10, 15, and 20 K $min^{-1}$. The degree of conversion ($\alpha$) was calculated according to:

$$\alpha = \frac{\Delta H_T}{\Delta H_{total}}$$

where $\Delta H_T$ is the cumulative reaction enthalpy released up to temperature T and $\Delta H_{total}$ is the total reaction enthalpy obtained by integrating the selected reaction region.

The Starink equation is expressed as:

$$\ln(\frac{\beta}{T_\alpha{}^{1.92}}) = C - 1.0008(\frac{E_a}{RT_\alpha})$$

where β is the heating rate (K $min^{-1}$), $T\alpha$ is the absolute temperature (K) at a specified conversion, $E_a$ is the apparent activation energy (J $mol^{-1}$), R is the universal gas constant (8.314 J $mol^{-1}$ $K^{-1}$), and C is a constant. For each fixed $\alpha$, $E_a$ was calculated from the slope of the linear relationship between ln(β/$T_\alpha$1.92) and 1/$T_\alpha$:

$$E_a = -\text{Slope} \times \frac{R}{1.0008}$$

As shown in Figure S1b and Table S1, the Starink plots exhibited moderate-to-good linearity over $\alpha$ = 0.1–0.9, with $R^2$ values ranging from 0.875 to 0.972. The apparent activation energy decreased from 87.90 kJ $mol^{-1}$ at $\alpha$ = 0.1 to 24.86 kJ mol–1 at $\alpha$ = 0.9, with an average value of approximately 46.06 kJ $mol^{-1}$.

The variation in $E_a$ with conversion suggests that the effective reaction barrier changes as curing proceeds. However, because the melting transition partially overlaps with the broad curing thermal event, baseline selection and conversion determination involve uncertainty, particularly at high conversion. Therefore, these activation-energy values should be regarded as approximate kinetic descriptors of the isolated LCE/DDM subsystem rather than precise kinetic parameters for the complete commercial potting formulation. Nevertheless, the results provide supplementary evidence of a temperature-dependent reaction between LCE and DDM.

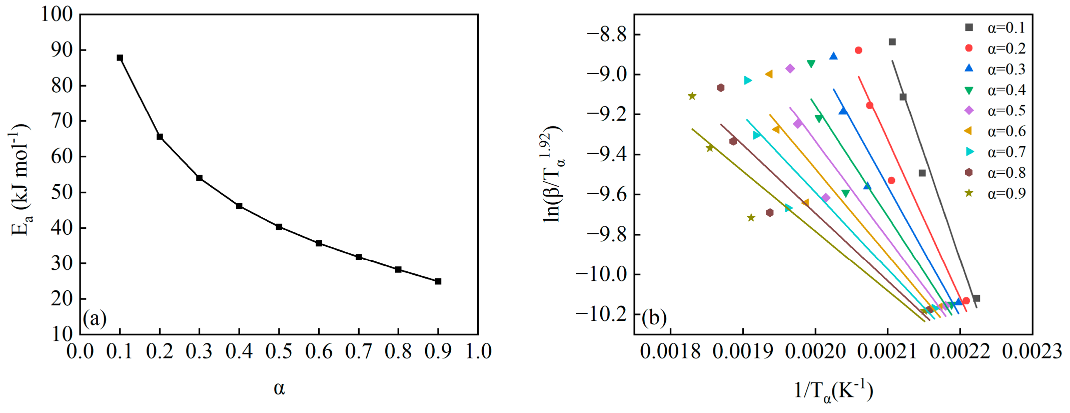


**Figure S1.** Curing kinetic analysis of the isolated LCE/DDM subsystem: (a) apparent activation energy

($E_a$) as a function of conversion ($\alpha$); (b) Starink plots of $\ln(\beta/T_{\alpha}1.92)$ versus $1/T_{\alpha}$ at $\alpha$ = 0.1–0.9.

**Table S1.** Starink kinetic parameters of the LCE/DDM subsystem at different degrees of conversion.

| $\alpha$ | Slope | $E_a$ (kJ mol$^{-1}$) | $R^2$ |
|---|---|---|---|
| 0.1 | −10581.33 | 87.90 | 0.972 |
| 0.2 | −7894.97 | 65.59 | 0.948 |
| 0.3 | −6498.33 | 53.98 | 0.925 |
| 0.4 | −5553.76 | 46.14 | 0.907 |
| 0.5 | −4856.69 | 40.35 | 0.890 |
| 0.6 | −4304.19 | 35.76 | 0.875 |
| 0.7 | −3827.48 | 31.80 | 0.875 |
| 0.8 | −3395.88 | 28.21 | 0.882 |
| 0.9 | −2992.88 | 24.86 | 0.892 |

The apparent activation energy was calculated using $E_a = -\text{slope} \times R/1.0008$, where R = 8.314 J mol$^{-1}$ K$^{-1}$.

***Section S2.*** *Evolution of Storage and Loss Moduli During Rheological Measurements*

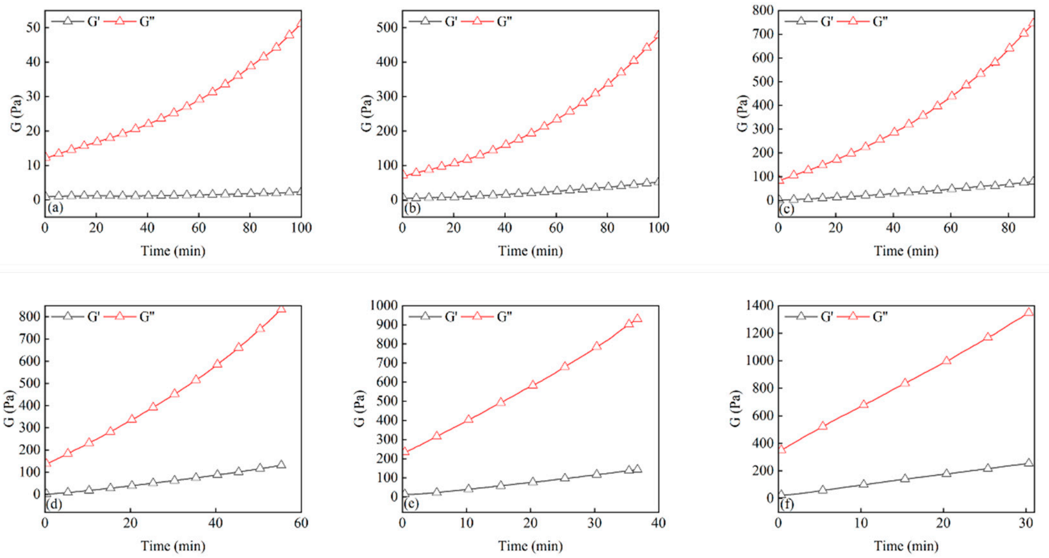


**Figure S2.** Time-dependent storage modulus (G′) and loss modulus (G″) of the LCE-modified potting compounds measured at 25 °C, 1 Hz, and 1.0% strain: (a) 0% LCE, (b) 10% LCE, (c) 20% LCE, (d) 30% LCE, (e) 40% LCE, and (f) 50% LCE.

Figure S2 presents the time-dependent storage modulus (G′) and loss modulus (G″) of all formulations under the rheological testing conditions used in this study. For all samples, G″ remained higher than G′ throughout the monitored time window, indicating that the viscous response dominated under the selected conditions. Both moduli changed continuously with time. Because a strain-sweep test was not performed, these curves do not independently establish that the measurements were conducted within the linear viscoelastic region and are provided only as complementary information for the comparative rheological measurements.

***Section S3.*** *SEM–EDS Elemental Mapping of Representative Fractured Surfaces*

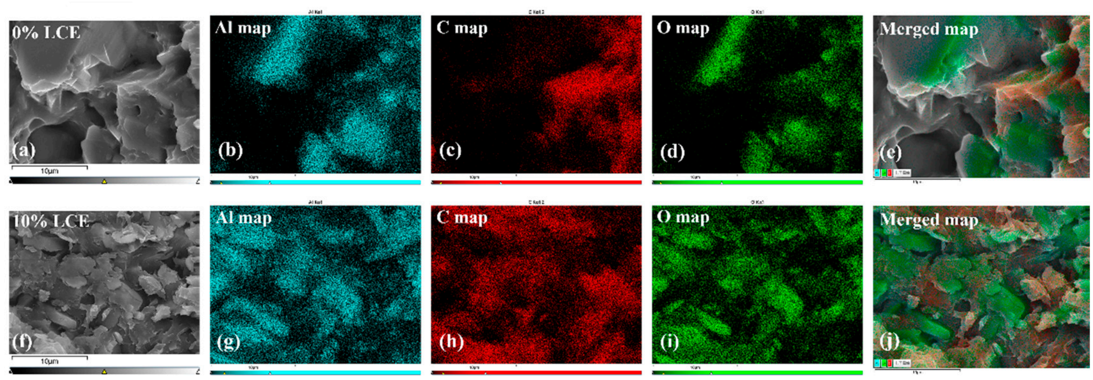


**Figure S3**. SEM images and corresponding Al, C, O, and merged elemental maps of representative fractured surfaces of the 0% and 10% LCE samples.

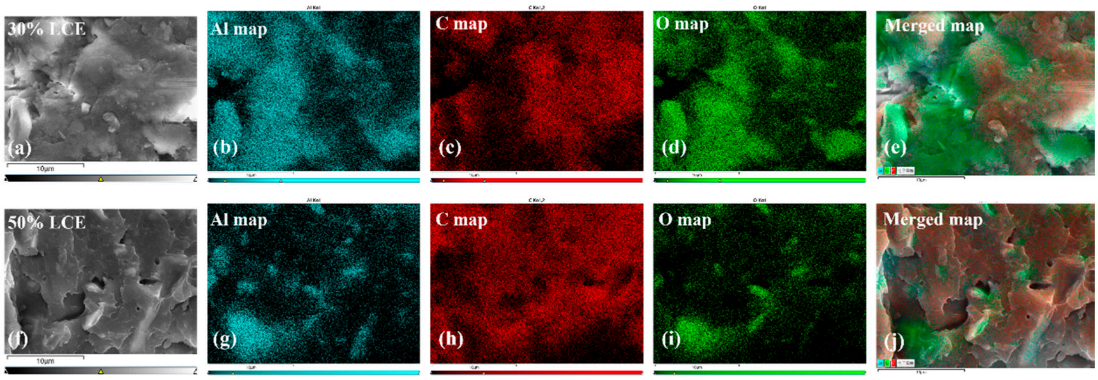


**Figure S4**. SEM images and corresponding Al, C, O, and merged elemental maps of representative fractured surfaces of the 30% and 50% LCE samples.
